# Growth‐Coupled Evolutionary Pressure Improving Epimerases for *D‐*Allulose Biosynthesis Using a Biosensor‐Assisted In Vivo Selection Platform

**DOI:** 10.1002/advs.202306478

**Published:** 2024-02-02

**Authors:** Chao Li, Xin Gao, Huimin Li, Tong Wang, Fuping Lu, Hui‐Min Qin

**Affiliations:** ^1^ Key Laboratory of Industrial Fermentation Microbiology of the Ministry of Education Tianjin Key Laboratory of Industrial Microbiology National Engineering Laboratory for Industrial Enzymes College of Biotechnology Tianjin University of Science and Technology Tianjin 300457 P. R. China

**Keywords:** biosensors, crystal structure analysis, *D‐*allulose 3‐epimerase, growth‐coupled selection platform, protein engineering

## Abstract

Fast screening strategies that enable high‐throughput evaluation and identification of desired variants from diversified enzyme libraries are crucial to tailoring biocatalysts for the synthesis of *D‐*allulose, which is currently limited by the poor catalytic performance of ketose 3‐epimerases (KEases). Here, the study designs a minimally equipment‐dependent, high‐throughput, and growth‐coupled in vivo screening platform founded on a redesigned *D‐*allulose‐dependent biosensor system. The genetic elements modulating regulator PsiR expression levels undergo systematic optimization to improve the growth‐responsive dynamic range of the biosensor, which presents ≈30‐fold facilitated growth optical density with a high signal‐to‐noise ratio (1.52 to 0.05) toward *D‐*allulose concentrations from 0 to 100 mm. Structural analysis and evolutionary conservation analysis of *Agrobacterium sp*. SUL3 *D‐*allulose 3‐epimerase (ADAE) reveal a highly conserved catalytic active site and variable hydrophobic pocket, which together regulate substrate recognition. Structure‐guided rational design and directed evolution are implemented using the growth‐coupled in vivo screening platform to reprogram ADAE, in which a mutant M42 (P38N/V102A/Y201L/S207N/I251R) is identified with a 6.28‐fold enhancement of catalytic activity and significantly improved thermostability with a 2.5‐fold increase of the half‐life at 60 °C. The research demonstrates that biosensor‐assisted growth‐coupled evolutionary pressure combined with structure‐guided rational design provides a universal route for engineering KEases.

## Graphical abstract

A growth‐coupled, high‐throughput screening platform is established using a redesigned *D*‐allulose‐dependent biosensor for engineering ketose 3‐epimerases (KEases). Structure‐guided rational design and directed evolution are performed to remodel the ADAE active sites and hydrophobic pocket for enhanced catalytic performance for the synthesis of D‐allulose. This approach offers a paradigm for the construction of biosensor‐assisted, growth‐coupled screening platforms.

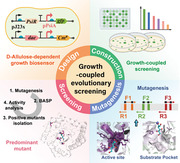

## 1 Introduction

*D‐*Allulose is a low‐calorie, rare ketohexose that is widely considered a prospective functional ingredient with multiple health‐beneficial physiological properties.^[^ ^1^ ^]^ Given the limited bioavailability in intestinal absorption, *D‐*allulose exhibits a considerably reduced glycemic index and caloric value, while possesses 70% sweetness of sucrose, making it an intriguing generally recognized as safe (GRAS) sugar alternative for the food industry.^[^ ^2^ ^]^ Simultaneously, *D‐*allulose also exerts numerous beneficial bioactivities, i.e., enhancing glucose tolerance, diminishing fat accumulation, scavenging reactive oxygen species, as well as protecting neuronal function.^[^ ^3^ ^]^ These health‐promoting functions of *D‐*allulose make it a natural functional component with high‐value commercial applications in the pharmaceutical, nutraceutical, and food industries.

Biocatalytic strategies have been developed to synthesize *D‐*allulose using ketose 3‐epimerases (KEases), which efficiently catalyze the reversible isomerization between *D‐*fructose and *D‐*allulose with high substrate specificity under mild reaction conditions.^[^ ^4^ ^]^ Recent research focused on the identification and biochemical characterization of diversified KEases, thus offering abundant biocatalysts for the synthesis of *D‐*allulose.^[^ ^5^ ^]^ Nevertheless, their industrial applicability in biotransformation has been restricted by inadequate catalytic activity and operational stability under harsh industrial reaction circumstances.^[^ ^6^ ^]^ Consequently, extensive and time‐consuming protein engineering efforts founded on rational design and directed evolution are usually required to customize natural enzymes for the production of *D‐*allulose.^[^ ^7^ ^]^ Most prominently, identifying and isolating desired variants from large and comprehensive mutant libraries remains a challenge since each individual with different phenotypes needs to be evaluated, in which case high‐throughput screening techniques are paramount and highly desirable to accelerate directed evolution processes for tailoring target enzymes with desired properties.^[^ ^8^ ^]^ Dual‐enzyme screening methods (coupled with xylose isomerase or ribitol dehydrogenase) were exploited to determine the activity of the KEases, enabling efficient screening of mutants with improved activity and thermostability.^[^ ^9^ ^]^ Furthermore, single‐cell evaluation techniques have also been developed employing fluorescence‐activated cell sorting or droplet‐based microfluidic assays, allowing high‐throughput screening of KEase mutants with superior catalytic activity.^[^ ^10^ ^]^ However, these existing methods are either limited by their low throughput (chromatography‐reliant assays and colorimetric assays in microtiter plates) or require extensive expertise and expensive equipment (e.g., robotic platforms or microfluidic devices). These limitations have prevented a more widespread implementation of KEase enzymes in biotransformation. Consequently, a high‐throughput screening strategy requiring minimal equipment would be extremely desirable for engineering *D‐*allulose synthesis enzymes.

Growth‐coupled evolutionary pressures offer an attractive alternative tool for enzyme engineering, since they link the desired catalytic properties of enzyme to the survival of the mutants.^[^ ^11^ ^]^ Such selection approaches intrinsically represent simplicity and high throughput, as the desired mutants were selected by means of generated cell growth phenotypes, a simple signal output mode using the optical density in liquid culture or colony formation/size in solid culture, while automatically discarding non‐functional variants.^[^ ^12^ ^]^ Despite the fact that growth selection is an extremely effective approach, its great potential was not previously harnessed in hunting superior enzymes for *D‐*allulose synthesis. Indeed, the growth‐coupled selection techniques available so far tend to be narrowly applicable only to very specific types of biomolecules or properties on account of requiring linkage between the activity of the enzyme and the survival of the host cells.^[^ ^13^ ^]^ Pioneering research on growth‐coupled evolution frequently concentrated on targeting proteins that directly impart antibiotic resistance (e.g., β‐lactamases), which are linked to the expression of antibiotic resistance genes.^[^ ^14^ ^]^ Biosensors have emerged as promising alternative approaches to relieve throughput limitations in metabolic pathways and enzyme engineering.^[^ ^15^ ^]^ Prominently, genetically encoded, transcription factor‐based biosensors can transmit the input signal (target metabolite concentration) into an easily detectable output (e.g., fluorescence, antibiotic resistance, or growth phenotype), offering a promising solution to accelerate the identification of target mutants.^[^ ^16^ ^]^ Numerous transcription factor‐based biosensors with ligand specificity and dynamic responsive ranges are already identified in nature for sensing various small molecules and improving the production of diverse metabolites.^[^ ^15^, ^17^ ^]^ For instance, an arginine‐responsive biosensor was designed by using the transcription factor ArgR with the *sacB* gene as an output reporter module. This screening system was employed to screen the *L‐*arginine producing strains by monitoring intracellular *L‐*arginine‐dependent cell growth phenotype.^[^ ^18^ ^]^ Another transcriptional regulator CcdR was redesigned to develop an *L‐*cysteine‐responsive biosensor for monitoring intracellular *L‐*arginine concentrations and further harnessed to achieve direct evolution of the *L‐*cysteine biosynthetic pathway to modify *L‐*cysteine‐producing strains from the random mutagenesis library.^[^ ^19^ ^]^ Recent expansions in the *D‐*allulose‐responsive biosensor founded on the transcription factor PsiR and its cognate promoter pPsiA highlighted the initial proof‐of‐concept that the expression of the green fluorescent protein gene can be linked to KEase activity in *Escherichia coli*, which together provide insightful guidelines to associate growth phenotype with KEase activity by regulating the expression of antibiotic resistance genes using the genetic biosensor.^[^ ^10b^ ^]^

In the present study, we redesigned the *D‐*allulose‐responsive biosensor system for real‐time monitoring of *D‐*allulose using cell growth phenotype, in which the growth‐responsive dynamic range was systematically optimized by fine‐tuning the regulator expression levels. We further leveraged the responsive growth phenotype to develop a facile, high‐throughput, and low‐equipment‐dependent in vivo selection platform with tunable sensitivity and a wider growth dynamic range for engineering KEases under special conditions. As a proof‐of‐concept, the structure‐guided rational design and directed evolution of the *Agrobacterium sp*. SUL3 *D‐*allulose 3‐epimerase (ADAE) was implemented to obtain enzyme variants with enhanced catalytic properties using the growth‐based selection platform. Structural analysis elucidated that the mutations in the selected M42 variant reorganized the hydrogen bonding network at the active site and strengthened interchain interactions, which together contributed to improved catalytic activity and thermostability. Our research provides a promising high‐throughput screening tool without specialized equipment for tailoring suitable biocatalysts for the efficient synthesis of *D‐*allulose.

## 2 Results and Discussion

### 2.1 Design and Optimization of a *D‐*Allulose‐Dependent Growth Biosensor System

**Figure 1:**
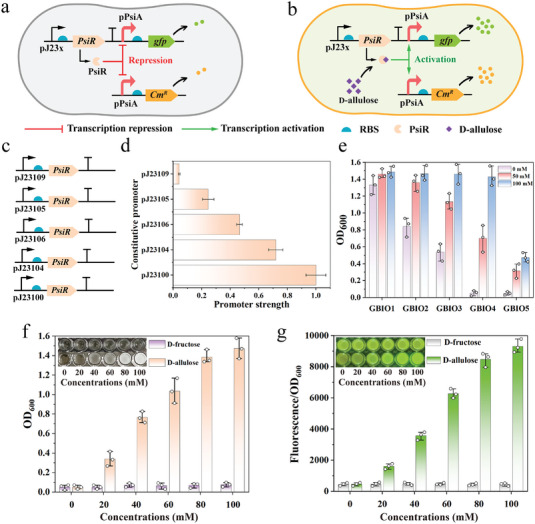
Development and optimization of the *D‐*allulose‐dependent growth biosensor for monitoring *D‐*allulose levels. a,b) Schematic diagram of the genetic architecture of the biosensor responsive to *D‐*allulose concentration in *E. coli*. c) The constructs of the signal‐response module in different versions. d) The five constitutive promoters with different transcription strengths used in this study. e) Cell growth of the GABIO1‐5 biosensor under chloramphenicol medium with various *D‐*allulose (0, 50, and 100 mm, respectively) at 12 h (*n* = 3). f) Quantitative analysis of the growth response of GBIO4 biosensor cells to *D‐*allulose and *D‐*fructose, respectively. The GBIO4 biosensor cells were cultivated in LB medium containing 0–100 mM *D‐*allulose or *D‐*fructose with 0.5 mg mL^−1^ chloramphenicol for 12 h (*n* = 3). g) Quantitative analysis of the fluorescence response of GBIO6 biosensor cells to *D‐*allulose and *D‐*fructose. The GBIO6 biosensor cells were cultivated in LB medium containing 0–100 mm *D‐*allulose or *D‐*fructose for 12 h (*n* = 3). Data are presented as mean values ± standard deviation (SD), *n* = 3 independent measurements.

To implement a generally applicable growth selection platform for screening KEases, we exploited a growth‐coupled biosensor system using an engineered transcriptional repressor PsiR from *Agrobacterium tumefaciens* (BBa_K2448025) with its regulatory promoter pPsiA to dynamically monitor the *D‐*allulose levels using cell growth phenotypes.^[^ ^10^ ^]^ The transcriptional repressor PsiR can bind to pPsiA promoter and then repress the transcription of downstream genes controlled by the pPsiA promoter in the absence of *D‐*allulose (**Figure** **1**a). By contrast, PsiR will preferentially bind to *D‐*allulose and then induce a conformational change, which results in PsiR dissociating from pPsiA and relieving the repressed transcription in the presence of *D‐*allulose (Figure 1b). A chloramphenicol resistance gene, *Cm^R^*, was placed downstream of the pPsiA promoter and used as an output signal in the reporter module to support visual observation for high‐throughput screening. Insufficient levels of *D‐*allulose presented in biosensor cells could lead to a decline and even stagnation in the transcription of the chloramphenicol resistance gene. Instead, increasing *D‐*allulose concentrations in the intracellular environment could relieve the repressed expression, thus restoring growth of the biosensor cells in the medium containing chloramphenicol. Therefore, the growth‐coupled biosensor system could commit to sensing intracellular *D‐*allulose levels and inducing the resistance gene *Cm^R^* expression, thus linking the *D‐*allulose concentration with the cell growth phenotype. For this purpose, the *D‐*allulose‐responsive repressor PsiR, expressed under the control of the constitutive promoters pJ23x (https://parts.igem.org/Part:BBa_J23110), was cloned into the pET22b plasmid as the signal‐response module, while the *Cm^R^* resistance gene, controlled by the pPsiA promoter, was integrated into the pET28a plasmid as an outputting signal in the reporter module (Figure 1a,b). Subsequently, the *D‐*allulose‐responsive biosensor system was introduced into *E. coli* BL21(DE3) to obtain the GBIOx biosensor strains for evaluating the growth‐coupled responsive performance.

GBIOx biosensors with broad responsive dynamic ranges represent important properties that make it possible to distinguish subtle differences of *D‐*allulose concentrations, significantly improving the efficiency of detection for phenotype screening. To obtain a functional biosensor system with a broad dynamic range of growth responses, we modulated the expression level of the transcription repressor PsiR using five constitutive promoters with different transcription strengths from weak to strong (pJ23109, pJ23105, pJ23106, pJ23104, and pJ23100) to fine‐tune the *D‐*allulose response range, resulting in the GBIO1‐5 biosensors (Figure 1c,d).

The growth phenotypes of GBIO1‐5 biosensor cells were tested in Luria‐Bertani (LB) media containing 0, 50, and 100 mm *D‐*allulose with 0.5 mg/mL chloramphenicol, respectively. As shown in Figure 1e, GBIO1‐3 biosensor cells are enough to restore growth in the presence of chloramphenicol without *D‐*allulose, indicating insufficient expression levels of the repressor PsiR controlled by the weak promoters pJ23109, pJ23105, and pJ23106, respectively. Conversely, the GBIO5 biosensor displayed a relatively low response sensitivity for *D‐*allulose on account of the excessive expression of the repressor PsiR controlled by the strong promoter pJ23100. Particularly, the GBIO4 biosensor, in which the expression level of the repressor PsiR was regulated by the constitutive promoter pJ23104, exhibited the best signal‐to‐noise ratio and the optimal dynamic range of growth responses corresponding to *D‐*allulose at different concentration levels. These findings indicated that the dynamic range of the biosensor was greatly affected by the expression level of the repressor PsiR, which was consistent with previous research.^[^ ^20^ ^]^ Continuous cell density (OD_600 nm_) profiling analysis was implemented to investigate dose‐response behaviors of the GBIO4 biosensor with gradient concentrations of *D‐*allulose (0, 20, 40, 60, 80, and 100 mm) as an input (Figure 1f). We observed a rank‐restored growth signal readout in the GBIO4 biosensor with the increase of *D‐*allulose concentrations, in which the GBIO4 biosensor presented ≈30‐fold facilitating growth with a high signal‐to‐noise ratio over a range of *D‐*allulose concentrations from 0 to 100 mm, highlighting the tight positive correlation between growth phenotypes and the *D‐*allulose concentrations. These findings revealed that fine‐tuning the expression level of the transcriptional repressor PsiR was favorable for improving the response capability of the biosensor. Importantly, the growth of the GBIO4 biosensor cells showed almost no response to *D‐*fructose, the substrate for *D‐*allulose biosynthesis, and was negligible above the background level even at a high *D‐*fructose concentration (Figure 1f). The result demonstrated that it is feasible to screen D‐allulose 3‐epimerase (DAEase) mutant libraries using this approach due to the excellent specificity of the biosensor for *D‐*allulose. Consistently, the GBIO4 biosensor's growth behavior in response to *D‐*allulose in LB solid medium mirrored the trend observed in LB fluid medium, in which the desirable growth behaviors demonstrated in both liquid and solid mediums support the selection flexibility for DAEase mutants library.

A common problem in growth‐coupled screening is that the cell growth phenotype may be unparallel to the catalytic activity of the target enzyme, particularly in distinguishing mutants with similar activity, as a result of the the complex determinants of cell growth. To circumvent this problem, a green fluorescence protein (GFP) gene controlled by the pPsiA promoter was placed downstream of the signal‐response module in the pET22b plasmid and applied as an orthogonal output signal in the reporter module, resulting in the GBIO6 biosensor. We observed a rank‐strengthened fluorescence signal readout in the GBIO6 biosensor cells at incremental *D‐*allulose concentrations, with a broad range of fluorescence induction fold changes (≈21‐fold at 100 mm *D‐*allulose) and induction specificity toward *D‐*allulose, consistent with the *D‐*allulose‐responsive growth phenotypes of the GBIO4 biosensor described above. Overall, a functional *D‐*allulose‐dependent growth‐coupled biosensor system was created with excellent dynamic responsiveness of growth phenotypes combined with a fluorescent signal readout responsive to *D‐*allulose, providing a versatile selective evolutionary pressure for improving the epimerization activity of KEases.

### 2.2 Design and General Procedure of the Biosensor‐Assisted In Vivo Selection Platform

**Figure 2:**
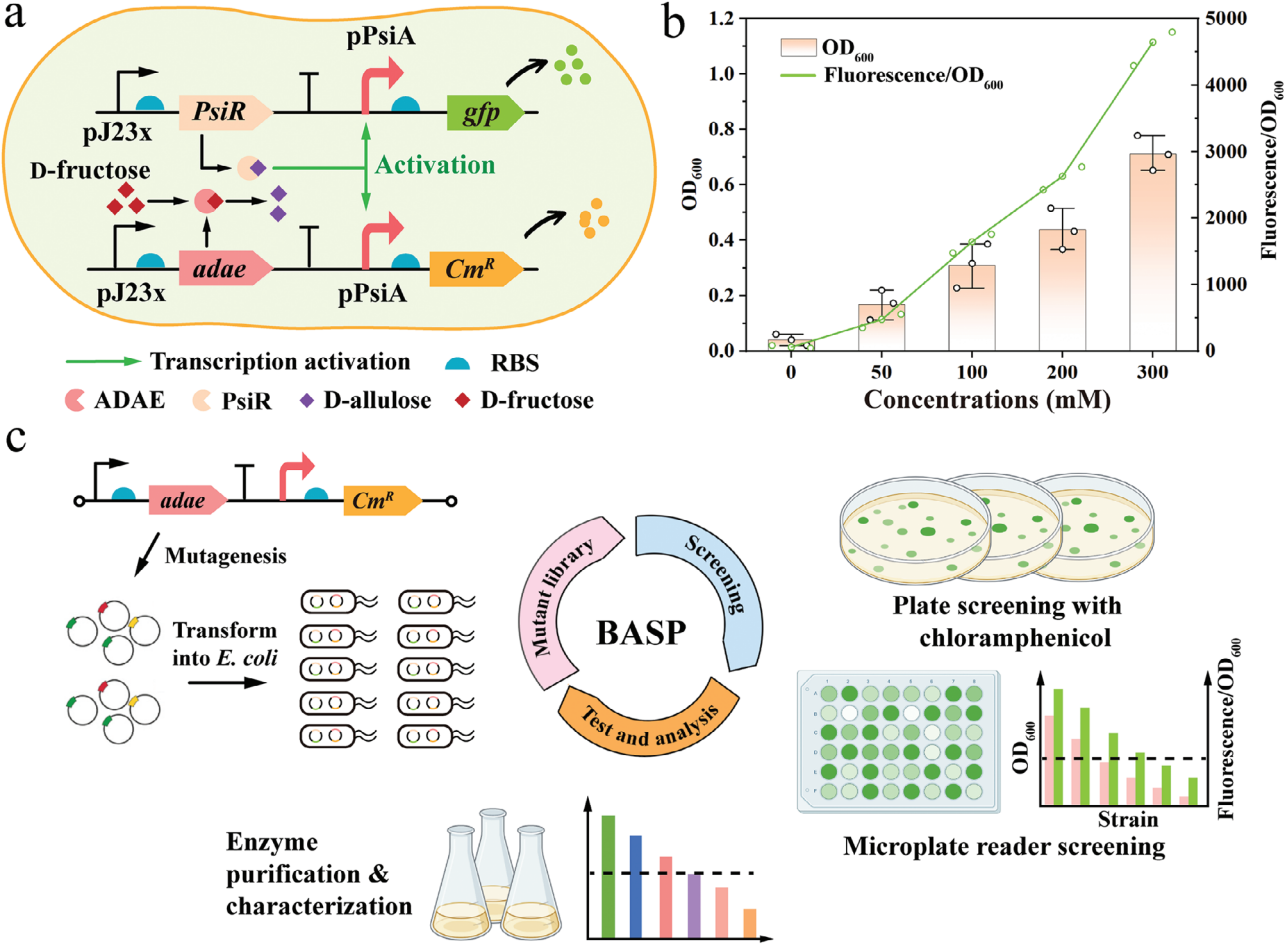
Biosensor‐assisted in vivo selection platform developed for the directed evolution of DAEase. a) Strategy for developing an enzyme‐screening system for DAEase. b) Quantitative analysis of the growth and fluorescence response of GBIO7 biosensor cells with 0–300 mm *D‐*fructose as substrate. GBIO7 biosensor cells were cultivated in LB fluid medium containing 0.5 mg mL^−1^ chloramphenicol with *D‐*fructose supplementation (0‐300 mm) for 12 h (*n* = 3). c) Schematic representation of the general procedure of biosensor‐assisted in vivo selection platform (BASP) for DAEase libraries. Mutagenesis of the gene encoding DAEase was cloned under a suitable promoter and then transformed into *E. coli* host cells. Selection in LB agar plates and liquid medium was performed by monitoring *D‐*allulose‐dependent cell growth phenotype and fluorescence intensity. Single colony of the DAEase variants was isolated, purified, and characterized. Data are presented as mean values ± SD, *n* = 3 independent measurements.

We sought to develop a high‐throughput in vivo selection platform using the *D‐*allulose‐dependent growth biosensor system to rapidly isolate the desired variants from DAEase libraries. For this purpose, we further integrated the DAEase expression cassettes under the control of constitutive pJ23x promoters into the GBIO6 biosensor system to obtain an enzyme‐screening system (**Figure** **2**a). The constitutive promoters with different strengths described above were employed to fine‐tune the expression levels of DAEase and thereby regulate the selection pressure, since the starting target enzyme with inferior activity requires a strong promoter to facilitate the conversion of sufficient *D‐*allulose to restore the growth of the candidate colonies. Instead, an intermediate‐ or low‐strength expression cassette guarantees the proliferation of cells bearing only the most active variants if a template with moderate or excellent activity has been presented in the starting strain.

An *Agrobacterium sp*. SUL3 DAEase (ADAE) with excellent thermostability and intermediate activity as expressed using the strong promoter pJ23100 was recruited to construct the enzyme‐screening system, resulting in the GBIO7 biosensor. Subsequently, the GBIO7 biosensor cells were cultivated at 37 °C for 12 h in LB medium containing 0.5 mg mL^−1^ chloramphenicol and different concentrations of *D‐*fructose (0–300 mm) as the substrate. As shown in Figure 2b, the expression of epimerization‐active ADAE facilitated GBIO7 biosensor cells growth in the presence of *D‐*fructose, which alone did not restore growth, accompanied by a strong positive correlation between the fluorescence intensity and *D‐*fructose concentration (Figure 2b). These results demonstrated that *D‐*allulose could be synthesized by ADAE using *D‐*fructose as substrate in the GBIO7 biosensor system, which in turn derepressed the expression of the *Cm^R^* and *gfp* genes controlled by the pPsiA promoter. These findings suggested that the constructed GBIO7 biosensor system is a workable tool for assessing the isomerization activity of DAEase and screening mutants with improved performance.

Given the preliminary GBIO7 biosensor enzyme‐screening system, the general procedure of a biosensor‐assisted in vivo selection platform (BASP) was proposed for high‐throughput screening of desired mutants from DAEase libraries (Figure 2c), which was composed of four major steps: i) the DAEase gene libraries are generated using site‐directed mutagenesis or error‐prone PCR, cloned into the enzyme‐screening system, and introduced into *E. coli* BL21(DE3) to generate the DAEase mutant library; ii) selection of mutants with large colony formation/size on LB agar plats under chloramphenicol selection pressure; iii) a second‐round screening of mutants in LB fluid medium under chloramphenicol selection pressure using a microplate reader; iv) the mutant strains outperforming control cells in terms of growth and fluorescence intensity are selected for sequencing and the ADAE variant genes are cloned into a single plasmid expression system for purification and activity characterization.

### 2.3 Evaluation of the Biosensor‐Assisted In Vivo Selection Platform for DAEase Screening

**Figure 3:**
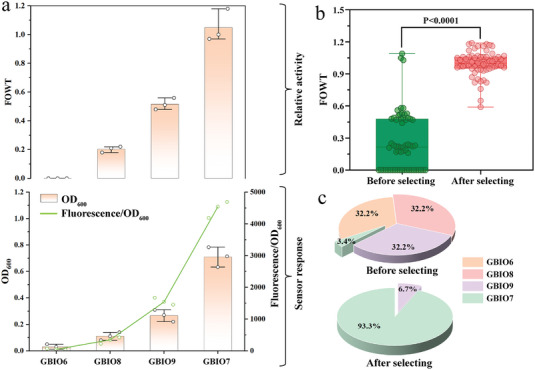
Evaluation of the biosensor‐assisted in vivo selection platform for DAEase library. a) Top: Relative activity of GBIO6‐9 cells (fold‐over GBIO7, FOWT). Bottom: Quantitative analysis of the growth and fluorescence response of GBIO6‐9 cells with 300 mM *D‐*fructose as substrate (*n* = 3). b) Enrichment demonstration of designed ADAE library. Boxplots represent the FOWT activity of cells from different populations (*p* < 0.0001). Before selecting, *n* = 60 independent colonies from the designed ADAE library; after selecting, *n* = 60 independent colonies from selected cells. c) The percentage of GBIO6‐9 cells in different populations, respectively. Data are presented as mean values ± SD, *n* = 3 independent measurements. Statistical significance was evaluated by the two‐tailed Student's *t*‐test.

We next evaluated the cell growth phenotypes and fluorescence response in strains with different ADAE expression levels using the biosensor‐assisted in vivo selection platform to benchmark the screening feasibility. Instead of using completely inactive control cells (GBIO6 without the DAEase expression cassette), we employed strains with reduced ADAE expression than GBIO7 to test BASP selection efficiency. For this purpose, we created the GBIO8 and GBIO9 strains expressing ADAE at relatively low levels controlled by two constitutive promoters, pJ23105 and pJ23106, respectively, showcasing inferior catalytic activities toward *D‐*fructose (**Figure** **3**a). Subsequently, the GBIO6‐9 biosensor cells were separately cultivated at 37 °C for 12 h in LB medium containing 0.5 mg mL^−1^ chloramphenicol and 300 mm *D‐*fructose as the substrate. As depicted in Figure 3a, the growth trend and fluorescence response of strains aligned well with the expressed DAEase activity. This indicated that the quantifiable growth phenotype and fluorescence signal can distinguish candidate enzymes with improved catalytic performance using this BASP platform. Meanwhile, increasing DAEase activity of stains results in even superior colony formation/size, allowing them to be more readily and reliably selected on LB agar plates under chloramphenicol selection pressure (Figure S2, Supporting Information).

Furthermore, an artificially mixed ADAE library was generated by combining the GBIO7/GBIO6/GBIO8/GBIO9 strains with 1:10:10:10 cell ratios, and screened using the BASP platform to evaluate the selection efficiency. The cell mixtures were spread on LB agar plates containing 300 mm *D‐*fructose as substrate with or without 0.5 mg mL^−1^ chloramphenicol as the selection pressure (Figure S3, Supporting Information). The results showed that only the cells with high ADAE activity can survive and grow in LB agar plates under chloramphenicol selective pressure, suggesting an intrinsic connection between growth selection and high‐throughput because only the desired highly active variants, instead of every variant, are detectable in directed evolution campaigns, underscoring the expediency of high‐performance phenotype screening. Following the selection using this BASP platform in the cell mixtures, significant enrichment of the highly active GBIO7 cells from the ADAE library was achieved (before selecting versus after selecton, *p* < 0.0001), in which cells with lower cell density and fluorescence intensity than GBIO7 cells were discarded to accumulate cells with optimal activity (Figure 3b). Encouragingly, we observed an ≈27‐fold enrichment for GBIO7 cells following this single round of BASP selection (Figure 3c), highlighting that the biosensor‐assisted in vivo selection platform was feasible for isolating predominant DAEase variants in large‐scale screening experiments.

### 2.4 Structural Analysis and Identification of Mutation Sites in ADAE

**Figure 4:**
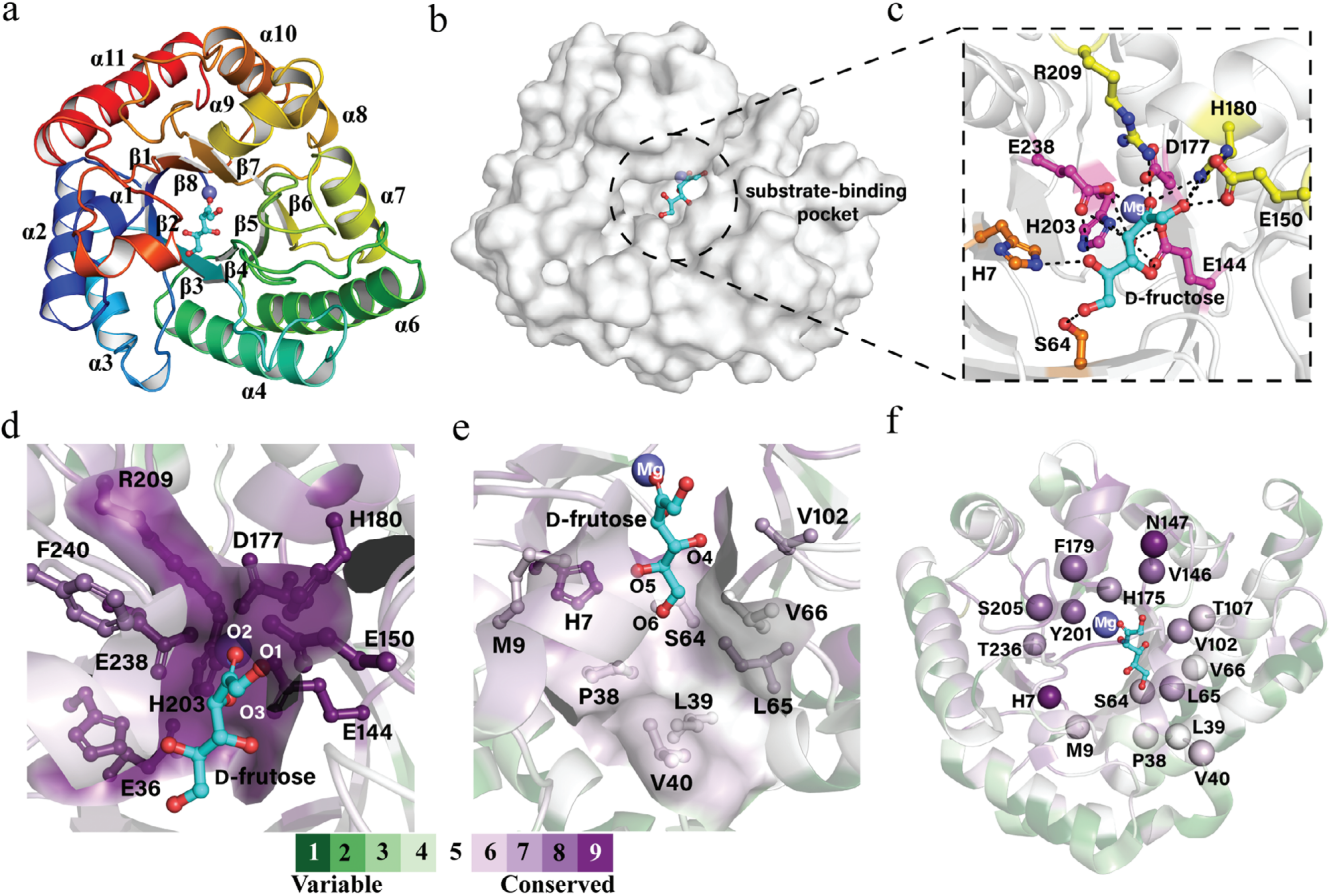
Structural analysis and identification of mutation sites in ADAE. a) Overall crystal structure of ADAE. b) Surface representation of the overall structure of ADAE. c) Enlarged view of the catalytically active pocket. The catalytic residues are shown as pink sticks. d,e) Evolutionary conservation analysis of ADAE structure in the catalytic active site (d) and hydrophobic substrate‐binding pocket region using Consurf server (e). The coloring of each residue corresponds to its degree of conservation in 10% increments. f) Locations of the point mutations identified by structural analysis in the crystal structure of ADAE. The C_α_ atoms of the identified mutants were highlighted as spheres.

Using gene database mining, we identified a new DAEase from *Agrobacterium sp*. SUL3, ADAE (Genebank accession number: WP_052820585.1), which presented a specific activity of 44.5 U/mg and a catalytic efficiency (*k*_cat_/*K*_m_) of 0.39 s^−1^ mm ^−1^ toward *D‐*fructose. The phylogenetic tree constructed based on the amino acid sequence of ADAE, together with characterized DAEases, DTEases, and LREases from other organisms, revealed that ADAE was closely related to homologs from *Sinorhizobium fredii* (GenBank: ASY72161.1). Moreover, ADAE exhibited the highest amino acid sequence identity with *S. fredii* DAEase (62.77%) (Figure S4, Supporting Information). This percentage indicated the uniqueness of ADAE. To gain insights into the precise structural mechanisms that underlie its enzyme activity, we obtained the X‐ray crystal structures of ADAE alone and in complex with different ligands, including *D‐*fructose and *D‐*allulose (Table S3, Supporting Information). ADAE formed a tetramer (Figure S5a, Supporting Information), and the each subunit structure comprised a well‐defined (α/β)_8_ triose‐phosphate isomerase‐barrel domain, which is highly conserved among the KEase family (**Figure** **4**a). The catalytic tetrad (Glu144, Asp177, His203, and Glu238) was assembled in coordination with the Mg^2+^ ion‐binding motif to form the metal‐containing active site located inside the substrate‐binding pocket (Figure 4b,c). The interactions of the bound *D‐*fructose with the active site residues are localized mainly at positions O‐1, O‐2, and O‐3, through which the *D‐*fructose is stabilized and positioned by hydrogen bonding interactions with residues Glu144, Glu150, His180, His203, Arg209, and Glu236. Evolutionary conservation analysis of ADAE using the ConSurf server showed that these substrate/metal‐interacting residues are highly conserved in structural integrity or functionality, forming a relatively narrow and restrictive binding region ensuring the correct substrate orientation (Figure 4d).^[^ ^21^ ^]^ In addition, multiple sequence alignments also showed that the proposed active site residues were absolutely conserved in the DAEase family, suggesting their crucial functional roles in the catalysis process (Figure S6, Supporting Information). Consequently, eight neighboring residues adjoining these conserved residues were identified as the first modification cluster for systematic variation to improve the catalytic performance (Figure 4f). In contrast, no sequence‐specific interactions of the enzyme were observed with the O‐4, O‐5, and O‐6 positions, in which the O‐5 group showed a possible interaction with His7, while the O‐6 group received a hydrogen bond from Ser64 (Figure 4c). Since few specific interactions occurred between the enzyme and respective ligands at the O‐4, O‐5, and O‐6 positions in both complexes, ADAE loosely recognizes substrates at these positions, leading to a highly disordered structure for this part of the substrate (Figure S5b, Supporting Information). Meanwhile, the residues around the O‐4, O‐5, and O‐6 positions of *D‐*fructose were entirely variable, creating a hydrophobic pocket involved in substrate recognition (Figure 4e; Figure S6, Supporting Information). These observations proposed that structural variability in the hydrophobic region might directly affect the substrate specificity and affinity of ADAE. As result in, nine residues lining the hydrophobic pocket were selected as the second mutation cluster with the aim of identifying variants that better recognize the O‐4, O‐5, and O‐6 positions of *D‐*fructose (Figure 4f). Collectively, seventeen candidate residues located in two functional regions (the vicinity of the conserved active sites and a variabe hydrophobic pocket) were selected for systematic optimization to create functional variations with improved catalytic performance.

### 2.5 Growth Selection‐Assisted Evolution of ADAE

**Figure 5:**
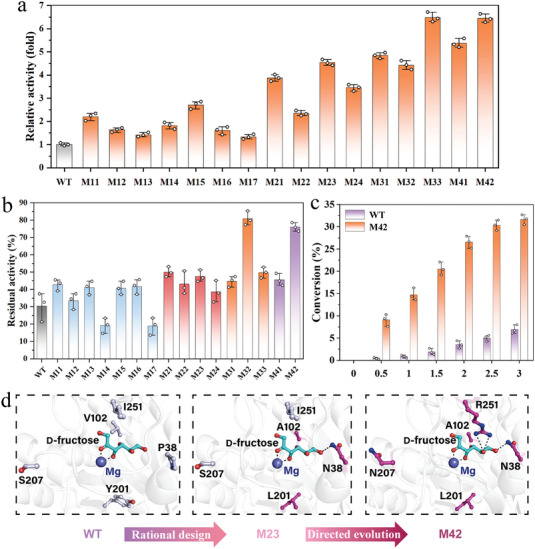
ADAE variants discovered using biosensor‐assisted in vivo selection. a) Relative specific activity of ADAE variants identified from selection. The enzyme activity of the WT ADAE was defined as 1.0 (*n* = 3). b) Relative residual activity of ADAE variants before and after heat treatment at 70 °C for 1 h. The initial activity of each sample was defined as 100% (*n* = 3). c) Time course of the synthesis of *D‐*allulose from *D‐*fructose using purified M42 variant and WT ADAE (*n* = 3). d) Variants of ADAE compared with the ADAE wild type. Variant M23 was created by structure‐guided rational design, while M42 was further generated from subsequent rounds of random mutagenesis. *D‐*Fructose was shown as cyan sticks, while Mg^2+^ was represented as a blue sphere. The mutation sites were shown as pick sticks. Data are presented as mean values ± SD, *n* = 3 independent measurements.

We sought to leverage the high‐throughput growth selection platform to improve the catalytic performance of the existing ADAE. Site‐saturation mutagenesis was conducted by randomizing all proposed candidate residues using NNK degenerate codons (Library 1, ≈3.4 × 10^2^ theoretical variants). The ADAE libraries were constructed using GBIO9 biosensor cells bearing the medium‐strength promoter pJ23106, and the resultant library quality was determined by growth experiments without chloramphenicol pressure followed by sequencing, which yielded ≈5 × 10^3^ independent transformants, ensuring thorough coverage of the theoretical diversity with tenfold oversampling. Selection was performed on LB agar plates containing 300 mm *D‐*fructose with 500 µg mL^−1^ chloramphenicol as the selection pressure. Following the preliminary plate screening, mutants with a larger colony size were picked for cultivation in 24 deep‐well plates in parallel to the selection pressure in plates, after which the cell growth and the relative fluorescence intensities of the isolated mutants were analyzed with GBIO9 cells harboring wild‐type ADAE as a negative control. Following three rounds of screening, the top 20 mutants with robust growth and higher fluorescence intensity were selected for DNA sequencing and further catalytic performance evaluation using purified enzyme. In this process, ADAE variants with poor thermostability were also discarded to preferentially obtain optimal mutations circumventing poor thermostability. A total of seven ADAE variants (M11 to M17) located at five sites with enhanced specific activity toward *D‐*fructose were identified (58.7–122.8 vs 44.5 U mg^−1^), including P38N, V102A, V102I, T107N, Y201L, Y201V, and T236K (**Figure** **5**a; Table S4, Supporting Information). Importantly, except for M14 (T107N) and M17 (T236K), the identified variants turned out to have much more robust thermostability, with ≈34.5–43.7% residual activity remaining after heat treatment for 1 h at 70 °C, which is significantly higher than its parent enzyme (32.2% residual activity) (Figure 5b). The single mutation Y201L resulted in a more pronounced improvement of catalytic activity than the other beneficial mutations, and thus was selected for further combinatorial mutagenesis. The M21 variant (Y201L/P38N) displayed a further improvement of specific activity to 172.2 U/mg (3.87‐fold over the wild type). Moreover, the M23 mutant (P38N/V102A/Y201L) exhibited an overall 4.52‐fold increase of specific activity over the wild type while maintaining robust thermostability with 46.6% residual activity remaining after heat treatment for 1 h at 70 °C (Figure 5a; Table S4, Supporting Information). By contrast, the combinatorial mutants M22 (P38N/T107N/Y201L) and M24 (P38N/V102A/Y201L/T236K) exhibited reduced enzyme activity compared to their parents (M21 and M23, respectively). Thus, the T107 and T236K substitutions were not included in further in efforts to improve the catalytic activity. The kinetic parameters (*K*_m_ and *k*_cat_ values) were also determined for the M23 mutant and parent ADAE with *D‐*fructose as substrate (**Table** **1**). Notably, a markedly increased *k*_cat_ value was discovered for the purified variant M23 (*k*_cat_ = 93.2 ± 1.3 s^−1^ vs 22.9 ± 0.5 s^−1^), which contributed to a higher catalytic efficiency (*k*_cat_/*K*_m_ = 2.43 s^−1^ mm ^−1^) than the parent ADAE (*k*_cat_/*K*_m_ = 0.39 s^−1^ mm ^−1^).

**Table 1:** Kinetic data of WT ADAE and its variants toward *D‐*fructose.

Enzymes	Mutations	*K*_m_ [mm]	*k*_cat_ [s^—1^]	*k*_cat_/*K*_m_ [s^−1^mm ^−1^]
WT	None	59.3 ± 2.5	22.9 ± 0.5	0.39
M23	P38N/V102A/Y201L	38.4 ± 3.2	93.2 ± 1.3	2.43
M42	P38N/V102A/Y201L/S207N/I251R	26.1 ± 3.7	95.7 ± 2.2	3.67

All experiments were performed in triplicate, and the results were expressed as the means ± standard deviation (SD).

We next leveraged BASP‐based directed evolution to search a broader protein sequence space using random mutagenesis to identify additional beneficial mutations on the basis of the M23 mutant. The library was introduced into the GBIO8 strain with the weak pJ23105 promoter to increase and fine‐tune the selection pressure, yielding ≈2×10^6^ independent transformants. Matching the selection operation procedure mentioned above, three variants (M31, M32, and M33) with additionally improved activity or thermostability were identified with accumulated T100E, S207N, or I251R mutations, respectively (Figure 5a; Table S4, Supporting Information). Among them, the quadruple variant M33 (P38N/V102A/Y201L/I251R) exhibited the highest specific activity of 286.6 U mg^−1^ toward *D‐*fructose (6.3‐fold over the wild type), while variant M32 showed markedly improved stability compared to its precursor M23 with 79.9% residual activity remaining after heat treatment for 1 h at 70 °C (Figure 5a; Table S4, Supporting Information). Furthermore, the combination mutant, designated M42, was constructed by introducing the S207N substitution into M33, which resulted in excellent activity similar to M32 while maintaining robust thermostability, with 75.7% residual activity remaining after heat treatment for 1 h at 70 °C. Instead, the combination mutants M41 (P38N/T100E/V102A/Y201L/I251R) showed decreased enzyme activity compared to its parent M33. Analysis of kinetic parameters revealed that variant M42 presented a 9.41‐fold increase of catalytic efficiency compared with the WT ADAE (*k*_cat_/*K*_m_ = 3.67 s^−1^ mm ^−1^ vs 0.39 s^−1^ mm ^−1^) (Table 1).

### 2.6 Characterization of ADAE and the M42 Variant Obtained through Growth‐Coupled Selection

*D‐*Fructose was used as the substrate to investigate the biochemical characteristics of WT ADAE and the M42 variant acquired using the BASP selection platform (Figure S7, Supporting Information). Both WT ADAE and the quintuple M42 variant showed maximum activity in PBS buffer pH 8.0, whereas the M42 variant continued to function well throughout a wider pH range (pH 6.5—11.0) than WT ADAE (pH 7.0–9.0) (Figure S7a, Supporting Information). Meanwhile, both WT ADAE and the M42 variant showed optimal activity at 70 °C, according to the temperature‐activity curve. (Figure S7b, Supporting Information). Importantly, M42 showed much better thermostability with a 2.5‐fold increase in half‐life at 60 °C, reaching 5.0 h (Figure S7c, Supporting Information), illustrating its potential for commercial production of *D‐*allulose. In addition, metal ions including Mg^2+^, Co^2+^, and Mn^2+^ improved its activity by 1.75‐, 1.72‐, and 1.28‐fold, respectively (Figure S7d, Supporting Information). During ADAE‐catalyzed *D‐*allulose synthesis, the best variant M42 afforded 32.2% conversion in only 3.0 h with a volumetric productivity of 53.7 g/(L∙h), whereas the wild‐type ADAE only yielded 6.8% conversion with a volumetric productivity of 11.3 g/(L∙h) within the tested reaction time (Figure 5c). Previously, Chen et al. reported the *Thermoclostridium caenicola* DAEase variant Var3 produced 137.5 g L^−1^ (a yield of 27.5%) *D‐*allulose from 500 g L^−1^ *D‐*fructose with a space‐time yield of 17.2 g/(L∙h).^[^ ^22^ ^]^ Taken together, these results suggest that ADAE M42 obtained via structure‐guided rational design and directed evolution using the BASP platform has promising commercial value for the industrial production of *D‐*allulose.

### 2.7 Molecular Basis of the Improved Catalytic Performance of the M42 Variant

To elucidate the structural basis underlining the improved catalytic performance of the mutations, we scrutinized the structural changes between WT ADAE and the M42 variant using molecular dynamics simulation (Figure 5d). The structural model of M42 indicated that the substitutions P38N and I251R surrounding the substrate binding pocket formed new hydrogen bonds with O6 and O5 of *D‐*fructose, respectively, favorably orienting the substrate in the active‐site pocket (Figure S8, Supporting Information). This finding revealed that these enhanced hydrogen bonding interactions with *D‐*fructose are at least partly responsible for the higher catalytic efficiency of M42 toward *D‐*fructose. This can also explain the reduced *K*_m_ values for *D‐*fructose in variant M42 compared to the WT ADAE (Table 1). The original residue clusters (Glu36, Leu176, Tyr201, and Thr236) assembled near the active site of the enzyme formed an internal hydrogen bonding network spanning the β2, β6, β7, and β8 regions, which contributed to a tight conformation in this region (Figure S9a, Supporting Information). By contrast, hydrogen bonding interactions were only observed between Lue176 and Lue201 in the M42 variant, which was attributed to the Y021L substitution (Figure S9b, Supporting Information). Consequently, we speculated that the diminished internal hydrogen bonding network contributed to increased flexibility in the catalytic center due to the attenuated traction force between the β2 and β8 regions, which may facilitate the substrate in entering and binding the catalytic center, resulting in the enhanced catalytic activity of M42. Moreover, structural modeling suggested that the alternative S207N residue could create two new hydrogen bonds with the strictly conserved residues N182 and E185 on the dimeric interface, strengthening the inter‐subunit interactions and thus countering disintegration, which contributed to the improvements of both thermal and structural stability (Figure S10, Supporting Information). Particularly, the V102A mutation slightly expanded the space inside the pocket, thus enlarging the active‐site pocket's capacity to accommodate the substrate (Figure S11, Supporting Information). These findings suggested that the mutations in variant M42 not only reshaped the active‐site pocket architecture, resulting in an increase of the substrate binding affinity, but also strengthened the inter‐chain interactions at the tetramer interface, thus exhibiting improvements of both the catalytic activity and thermostability compared with the WT ADAE.

## 3 Conclusion

We developed a growth‐coupled, high‐throughput screening platform for engineering KEases using a redesigned *D‐*allulose‐dependent biosensor system, in which the growth‐responsive dynamic range of the biosensor was systematically optimized by fine‐tuning regulator expression levels. Mutant libraries of *Agrobacterium sp*. SUL3 *D‐*allulose 3‐epimerase (ADAE) were constructed via structure‐guided selection of mutation sites and directed evolution, in which the M42 variant (P38N/V102A/Y201L/S207N/I251R) was identified with a 6.28‐fold enhancement of enzyme activity and outstanding thermostability using the biosensor‐assisted in vivo selection platform. Structural analysis elucidates that precise reorganization of the active site hydrogen bonding network and strengthening of inter‐chain interactions together contributed to the improved catalytic performance. Our research offers a promising high‐throughput screening tool for biocatalyst customization for the efficient synthesis of *D‐*allulose.

## Conflict of Interest

The authors declare no competing interests.

## Author Contributions

F.L. and H.‐M.Q. designed the research. C.L., X.G., and H.L. performed the experiments. H.‐M.Q., C.L., and T.W. analyzed the data. H.‐M.Q. and C.L. wrote the manuscript. All authors have approved the final version of the manuscript.

## Supporting information

Supporting Information

## Data Availability

The data that support the findings of this study are available in the supplementary material of this article.
