# Supplementary material for: Growth‐Coupled Evolutionary Pressure Improving Epimerases for D‐Allulose Biosynthesis Using a Biosensor‐Assisted In Vivo Selection Platform
Source: Adv Sci (Weinh). 2024 Feb 2;11(14):2306478. doi: 10.1002/advs.202306478 (PMC11005681; doi:10.1002/advs.202306478)
Supplement: Supplementary file 1 — Supporting Information [file ADVS-11-2306478-s001.pdf]

ADVANCED SCIENCE
Open Access

Supporting Information

for *Adv. Sci.*, DOI 10.1002/advs.202306478

Growth-Coupled Evolutionary Pressure Improving Epimerases for *D*-Allulose Biosynthesis Using a Biosensor-Assisted In Vivo Selection Platform

*Chao Li, Xin Gao, Huimin Li, Tong Wang, Fuping Lu and Hui-Min Qin**

Supporting Information

Growth-Coupled Evolutionary Pressure Improving Epimerases for D-Allulose Biosynthesis Using a Biosensor-Assisted *In Vivo* Selection Platform

*Chao Li, Xin Gao, Huimin Li, Tong Wang, Fuping Lu, Hui-Min Qin**

Key Laboratory of Industrial Fermentation Microbiology of the Ministry of Education; Tianjin Key Laboratory of Industrial Microbiology, College of Biotechnology, Tianjin University of Science and Technology; National Engineering Laboratory for Industrial Enzymes, Tianjin 300457, P. R. China.

Email: huiminqin@tust.edu.cn.

1. Experimental Section

1.1. Strains and Reagents

All strains and plasmids utilized in this work are summarized in Table S1. Routine cloning was conducted using *E. coli* JM109. *E. coli* BL21 (DE3) was employed for target protein expression and as the starting strain for customizing biosensor strains. All chemicals utilized in this work were acquired from Sigma-Aldrich (Shanghai, China) at analytical grades. 2×Super Pfx MasterMix was applied for gene amplification and purchased from CWBIO (Taizhou, China). T4 DNA ligase and restriction endonucleases were obtained from Takara Biotechnology Co., Ltd. (Dalian, China). New England BioLabs (Beverley, MA, USA) provided the Stratagene

GeneMorph II random mutagenesis kit and the KOD-Plus-Mutagenesis Kit. All oligonucleotides were generated by AZENTA (Suzhou, China).

1.2. Construction of the D-Allulose-Dependent Growth Biosensor System

To construct the D-allulose-dependent growth biosensor system, the pJ23x-*psiR* gene fragments were synthesized and inserted into the pET22b plasmid between the *Nde*I and *Hin*dIII restriction sites, respectively, whereby a transcription terminator was inserted at the C-termini of *psiR* to avoid transcriptional noise. Simultaneously, the pPsiA promoter and chloramphenicol resistance gene fragments were synthesized and ligated by overlap-extension PCR using the primers pPsiA-Cm^R-U and pPsiA-Cm^R-D, resulting in the pPsiA-Cm^R gene fragment, which was inserted into the pET28a plasmid between the *Hin*dIII and *Xho*I restriction sites. Subsequently, *E. coli* BL21 (DE3) was co-transformed with the two plasmids systems to generate the GBIOx biosensors, in which five constitutive promoters pJ23x (pJ23109, pJ23105, pJ23106, pJ23104, and pJ23100) with different transcription strengths from weak to strong were selected to fine-tune the expression of PsiR (https://parts.igem.org/Part:BBa_J23110), resulting in the five biosensors designated GBIO1-5 (Table S1). Furthermore, the green fluorescent protein (GFP) gene fragment, *gfp*, was synthesized and ligated with the pPsiA promoter using overlap-extension PCR with the primers pPsiA-*gfp*-U and pPsiA-*gfp*-D to generate the pPsiA-*gfp* gene fragment, which was then inserted downstream of the pJ23104-*psiR* gene fragment to create the GBIO6 biosensor. To construct the enzyme-screening system for KEases, the *Agrobacterium* sp. SUL3 DAEase gene fragment, *adae*, was synthesized, fused with the constitutive promoters pJ23x, respectively, and subcloned upstream of the pPsiA-Cm^R gene fragment in the pET28a plasmid between the *Nco*I and *Hin*dIII restriction sites, whereby a transcription terminator was inserted at the C-termini of *adae* to avoid transcriptional noise. Three constitutive promoters pJ23x (pJ23105, pJ23106, and pJ23100) were recruited to regulate the expression of the *adae* gene, resulting in the GBIO8,

GBIO9, and GBIO7 biosensors, respectively (Table S1). The primers used for biosensor construction are listed in Table S2.

1.3. Characterization of the Biosensor's Dynamic Range

To characterize the created biosensor systems, biosensor strains were precultured in Luria-Bertani (LB) medium overnight at 37 °C with 100 μg/mL ampicillin and 50 μg/mL kanamycin. The resulting seed culture was diluted 100-fold using the same medium in 24-deep-well plates, which were filled with 3.5 mL per well of the diluted cell suspension containing gradient concentrations of D-allulose (0-100 mM) or D-fructose (0-300 mM) with 500 μg/mL chloramphenicol to determine the dose-response curve. Following incubation at 37 °C and 600 rpm for 12 hours, the cell density (OD_{600}) was measured using a conventional microplate reader (Neo2, BioTek, USA), and the fluorescence intensity was measured using a Synergy^{TM} HTX plate reader (BioTekVR Instruments, Inc.) at an excitation wavelength of 485/20 nm and an emission wavelength of 528/20 nm. The fluorescence values were normalized to the OD_{600}. All experiments were carried out in triplicate, and the data were presented as the means of three replicate experiments (n = 3).

1.4. Generation of ADAE Mutant Libraries Using Site-Directed Saturation and Random Mutagenesis

Saturation mutagenesis libraries of ADAE were constructed using the KOD-Plus-Mutagenesis kit following standard instruction, in which beneficial sites were replaced by the degenerate NNK codon (where N = A, G, C, or T and K = G or T). PCR reactions were conducted using pJ23106-adae-pPsiA-Cm^R-pET28a plasmid as the template and the primers devised and created by AZENTA listed in Table S2. The templates were digested from amplified PCR products using *Dpn*I before being ligated with T4 DNA ligase for 1 h at 16 °C. Subsequently, The ligated product and pJ23104-psiR-pPsiA-*gfp*-pET22b plasmid were co-transformed into *E. coli* BL21(DE3) competent cells to obtain a *site-directed saturation* mutagenesis library.

ADAE M1-3 was further subjected to error-prone PCR to generate an ADAE random mutagenesis library using the Stratagene GeneMorph II random mutagenesis kit following standard instructions. PCR reactions were conducted using pJ23x-adae-pPsiA-Cm^R-pET28a plasmid as the template and ADAE-F and ADAE-R primers created by AZENTA listed in Table S2. The mutation rate was adjusted by altering manganese ion concentrations with 1-4 mutations per 1000 base pairs. Following the digestion using corresponding restriction endonucleases, the amplified PCR products were inserted into the pJ23105-adae-pPsiA-Cm^R-pET28a plasmid using T4 ligase. Subsequently, the recombinant plasmid and pJ23104-psiR-pPsiA-gfp-pET22b plasmid were co-transformed into *E. coli* BL21(DE3) competent cells to obtain a random mutagenesis library.

1.5. Screening of ADAE Mutant Libraries Using the BASP Platform

The ADAE libraries with intermediate or weak constitutive promoters (pJ23106 or pJ23105) were constructed as described below. The ADAE libraries were introduced into corresponding competent biosensor cells, which were incubated at 37 °C with shaking for 1 hour. A small portion of the transformed cells was isolated to test the transformation efficiency, and the rest of the transformants were spread onto LB agar plates containing 50 μg/mL kanamycin and 100 μg/mL ampicillin with 300 mM D-fructose as substrate and 500 μg/mL chloramphenicol as selection pressure. GBIO7 cells containing wild-type ADAE or template variant with corresponding promoters were also spread onto LB agar plates as a control. Following incubation at 37 °C for 12 hours, the mutant strains with large and representative colonies as well as the control strains were picked and cultivated in 24 deep-well plates with 2.5 mL LB medium in parallel to the selection pressure in plates. Following incubation at 37 °C and 600 rpm for 12 hours, 200 μL cell of the culture from each strain was transferred into a 96-well plate to measure the cell densities and fluorescence intensities as described above. Sequencing was performed on

the mutant strains that outperformed control cells in terms of growth and fluorescence intensity. Subsequently, the ADAE variant genes were individually cloned into a single expression system in pET22b vector for purification and activity characterization as described below.

1.6. Cloning, Expression, and Purification of ADAE

The codon-optimized coding sequences of *Agrobacterium* sp. SUL3 ADAE (GenBank accession No. WP_052820585.1) and ADAE variants were subcloned into the pET22b plasmid between the *Nde*I and *Xho*I restriction sites, respectively. *E. coli* BL21(DE3) was transformed with the constructed pET22b plasmid, and then cultivated in LB medium with 100 g/mL ampicillin at 37 °C and 220 rpm until the OD600 reached 0.6-0.8. Subsequently, target enzymes were overexpressed at 16 °C for 16-18 hours with the addition of isopropyl-β-D-thiogalactopyranoside (IPTG) at a final concentration of 0.5 mM. Following centrifugation of the induced cells at 5,000 g for 15 min at 4 °C, the target enzymes were purified as in our previous study.[1] The purified target enzymes were dialyzed against 20 mM phosphate-buffered saline (PBS) (pH 7.5) for enzyme activity assays or 20 mM Tris-HCl (pH 8.0) used for crystallization. Sodium dodecyl sulfate polyacrylamide gel electrophoresis (SDS-PAGE) was used to evaluate the purified proteins, and the protein concentration was determined using a BCA assay kit (Solarbio, China).

1.7. Enzymatic Activity Assay

The catalytic activities of ADAE and its variants were determined by measuring the formation of D-allulose using D-fructose as substrate. A 1 mL reaction mixture was comprised of 1 μM purified target enzyme, 10 g/L D-fructose as substrate, and 1 mM Mg^{2+} in 20 mM PBS (pH 7.5). The reactions were carried out at 70 °C for 5 min, and then D-allulose synthesized by ADAE or its variants was measured using high-performance liquid chromatography (HPLC) following a 10-fold dilution. An Agilent 1260 HPLC system with an evaporative light-scattering detector (Agilent, USA) was used to analyze the samples. Chromatographic separation was performed

using a Prevail Carbohydrate ES column-W (5 μm, 4.6 × 250 mm, Agela Technologies, China). 75% acetonitrile was used as mobile phase to elute samples with a flow rate of 1 mL/min at 40 °C. The quantity of enzyme required to generate 1 μmol D-allulose each minute was defined as one unit of enzyme activity under standard assay conditions. Each experiment was performed in triplicate, and all data were shown as the mean of three replicate experiments (n = 3).

1.8. Characterization of ADAE

The reactions were performed using the standard activity assay protocol across a pH range of 5.0-11.0, pH 5.0-5.5 (sodium acetate buffer), pH 5.5-6.5 (4-morpholineethanesulfonic acid buffer), pH 7.0-8.0 (PBS buffer), pH 8.5-9.0 (Tris-HCl buffer), and pH 9.5-11.0 (3-(cyclohexylamino)-1-propanesulfonic acid buffer), to determine the effect of pH on ADAE and its variants. The optimum temperature of ADAE was measured from 45 to 85 °C under standard conditions. The thermostability of ADAE and its variants was investigated by incubating the purified protein (1 μM) at 60 °C for 6 h in 20 mM PBS buffer (pH 7.5), and the residual activity was tested at fixed intervals (0.5 h). The reactions were also conducted under standard conditions with 1 mM Ca^{2+}, Ni^{2+}, Zn^{2+}, Cu^{2+}, Co^{2+}, Mg^{2+}, Mn^{2+}, Ba^{2+}, and Fe^{2+} supplements, respectively, to investigate the effects of metal ions, in which the activity without the presence of metal ions was deemed to be 100%. Kinetic parameters (K_m and k_{cat}) of purified enzymes were calculated under standard conditions using continuous kinetic assays using 0-500 mM D-fructose as substrate. Prism 7.0 (GraphPad Software, La Jolla, CA, USA) was used to determine the kinetic parameters via non-linear regression. A 25-mL reaction mixture comprising 1 μM purified enzyme, 1 mM Mg^{2+}, and 500 g/L D-fructose was incubated under optimal conditions for the biosynthesis of D-allulose, and the reaction mixture was taken at intervals to test the formation of D-allulose using HPLC. All reactions were performed in three parallel experiments.

1.9. Crystallization, Data Collection, and Structure Determination

The sitting-drop vapor diffusion method was used to crystallize ADAE, in which the crystallization drops were obtained by growing from a 1:1 mixture of ADAE solution (10 mg/mL) and reservoir solution (0.2 mol/L Lithium sulfate monohydrate, 0.1 mol/L Tris pH 8.0, and 30% PEG 3,350) at 20 °C. Under the same conditions, 50 mM D-fructose and D-allulose were utilized with ADAE for the crystallization of the protein-ligand complex, respectively. A diffraction dataset was collected on beamline AR-NE3A at the Photon Factory (Tsukuba, Japan). The crystals were picked up with a nylon loop (Hampton Research, Aliso Viejo, CA) and directly flash-cooled in a nitrogen cryostream (-178 ℃) without cryoprotectant. The crystals were taken up using a nylon loop (Hampton Research, Aliso Viejo, CA), and immediately flash-cooled in a nitrogen cryostream (-178 °C) without the use of cryoprotectant. The XDS program was used to index, integrate, and scale the diffraction data. Using the crystal structure of SfDAE (PDB code: 7DZ2) as a search model,[2] all structures were solved using the molecular replacement approach in PHASER. The initial models were extended and modified manually in COOT,[3] and subsequently refinement was performed in PHENIX.[4] Crystallographic data were deposited in the PDB under accession codes 7ERN,7ERO, and 7ERM, respectively. Data collection and refinement statistics are presented in Table S3. All structural figures were generate using PyMOL (http://www.pymol.org/).

1.10. Statistical Analysis

GraphPad Prism 7.0 (GraphPad Software, La Jolla, CA, USA) was applied to analyzed experimental data obtained from at least three independent measurements. All the experimental date were presented as means ± standard deviation (SD). Statistical significance was evaluated by carrying out a two-sided Student's t-test between two groups, in which groups with $p < 0.05$ were considered to be statistically significant.

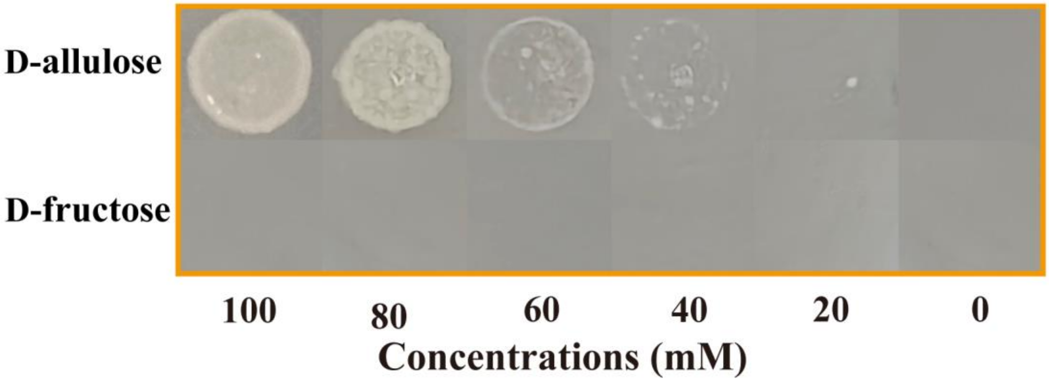

Figure S1. Validation of the growth response of GBIO4 biosensor cells to D-allulose and D-fructose under chloramphenicol selective pressure. The GBIO4 biosensor cells were cultivated in LB solid medium containing 0-100 mM D-allulose or D-fructose with 0.5 mg/mL chloramphenicol for 12 h, in which growth restoration was achieved with the introduction of D-allulose but not D-fructose.

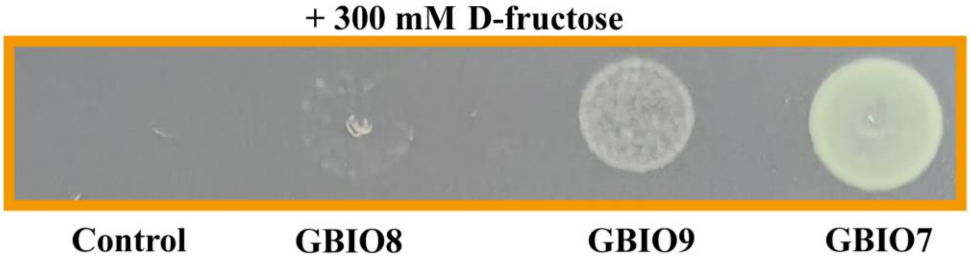

Figure S2. Validation of the growth response of GBIO7 biosensor cells under chloramphenicol selective pressure with 300 mM D-fructose as substrate. The GBIO7 biosensor cells were cultivated in LB solid medium containing 300 mM D-fructose and 0.5 mg/mL chloramphenicol for 12 h.

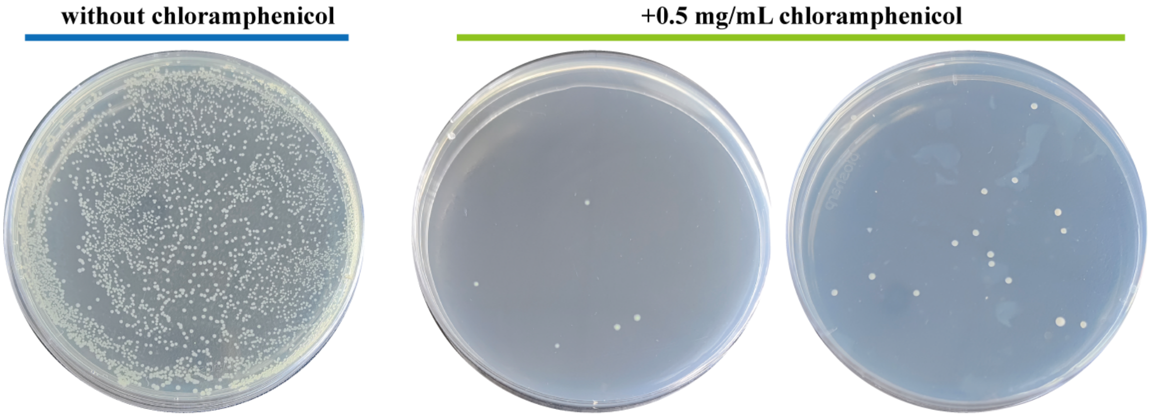

Figure S3. Validation of the growth and transformation efficiency of the artificially mixed ADAE library. The artificially mixed ADAE library was cultivated in LB solid medium containing 300 mM D-fructose with or without 0.5 mg/mL chloramphenicol for 12 h.

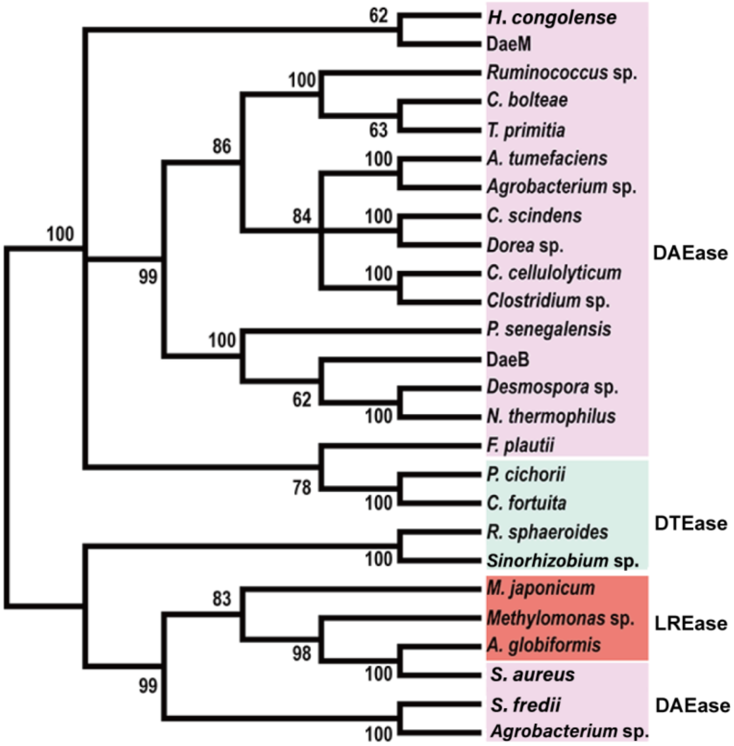

Figure S4. The phylogenetic tree of keto 3-epimerases from different strains. The microorganism origins with GenBank accession numbers were as follows: *Halanaerobium congolense* (WP_110301365.1), DaeM (QHD25651.1), *Ruminococcus* sp. (ZP_04858451.1), *Clostridium bolteae* (EDP19602.1), *Treponema primitia* (ZP_09717154.1), *Agrobacterium tumefaciens*

(AAK88700.1), *Agrobacterium* sp. (EGL65884.1), *Clostridium scindens* (WP_004607502.1), *Dorea* sp. (WP_022318236.1), *Clostridium cellulolyticum* (ACL75304.1), *Clostridiium* sp. (WP_014314767.1), *Paenibacillus senegalensis* (WP_010270828.1) DaeB (KYG89858.1), *Desmospora* sp. (WP_009711885.1), *Novibacillus thermophilus* (WP_077721022.1), *Flavonifractor plautii* (EHM40452.1), *Pseudomonas cichorii* (BAA24429.1), *Caballeronia fortuita* (WP_061137998.1), *Rhodobacter sphaeroides* (ACO59490.1), *Sinorhizobium* sp. (WP_069063284.1), *Mesorhizobium japonicum* (BAB50456.1), *Methylomonas* sp. (WP_064020855.1), *Arthrobacter globiformis* (BAW27657.1), *Staphylococcus aureus* (SQA09501.1), *Sinorhizobium fredii* (ASY72161.1), and *Agrobacterium* sp. SUL3 (WP_052820585.1).

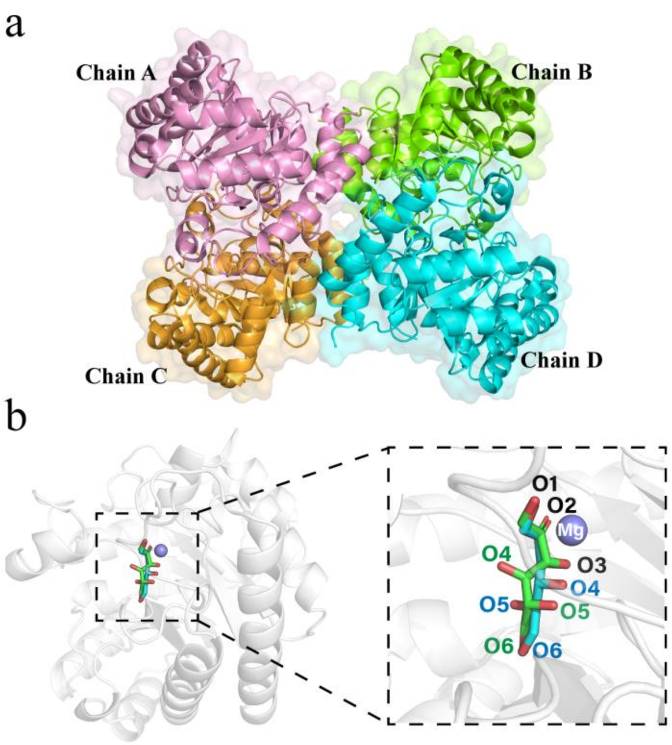

Figure S5. The crystal structures of SfDAE and its complex with D-fructose and D-allulose. (a)

The overall tetramer structure of ADAE. (b) Superposition of D-fructose and D-allulose molecules in the structure complex. D-Fructose and D-allulose were shown as sticks with the colors cyan and green, respectively. The Mg^{2+} was represented as a blue sphere.

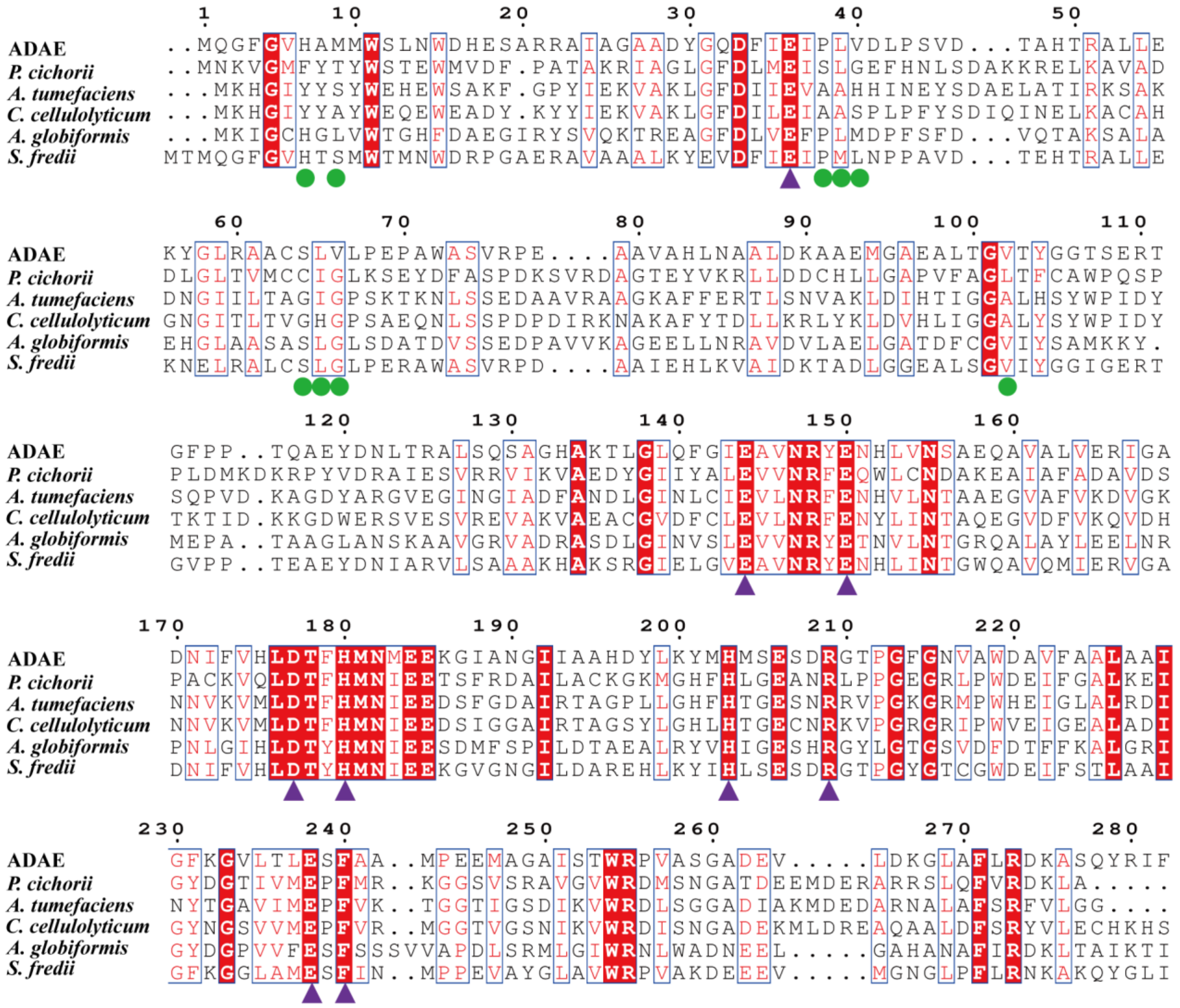

Figure S6. Multiple sequence alignments of DAEases from different strains. The triangle indicates the extremely conserved residues coordinating with the metal ion or the O-1, O-2, and O-3 of D-fructose in the active site, respectively. The circle indicates the variable residues lining the hydrophobic pocket.

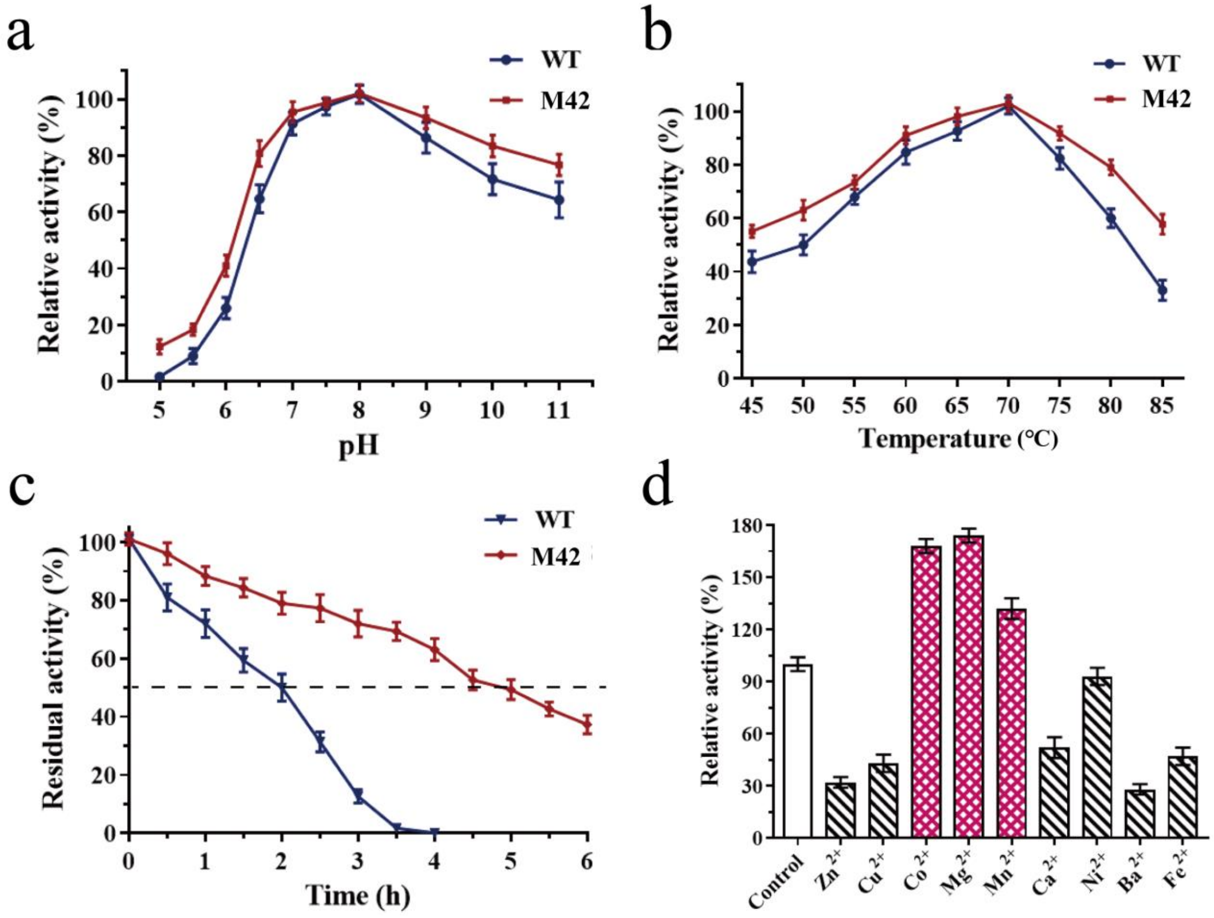

Figure S7. Biochemical properties of WT ADAE and the best variant M42. (a) pH dependence, (b) temperature dependence, (c) thermostability analysis of WT ADAE and the variant M42, (d) effect of metal ions on the activity of ADAE. Data are presented as mean values ± SD, *n* = 3 independent measurements.

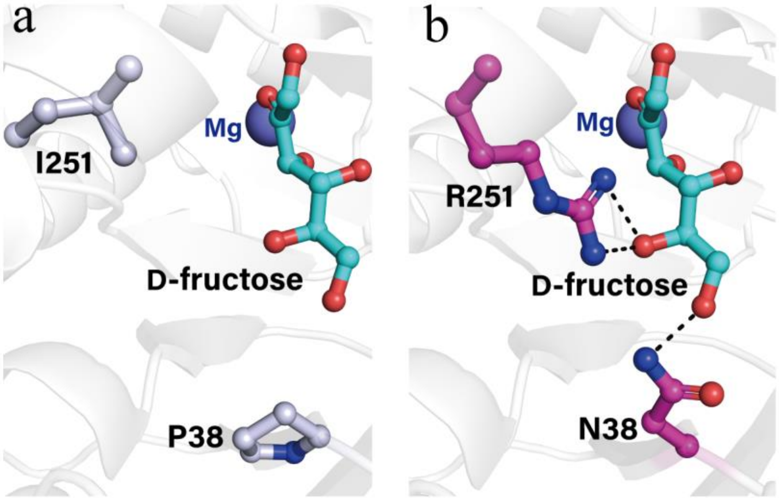

Figure S8. Structural representation of the substrate binding pocket of WT ADAE (a) and the variant M42 (b). D-fructose was shown as cyan sticks, while Mg^{2+} was represented as a blue sphere. Hydrogen bonds were shown as black dashed lines.

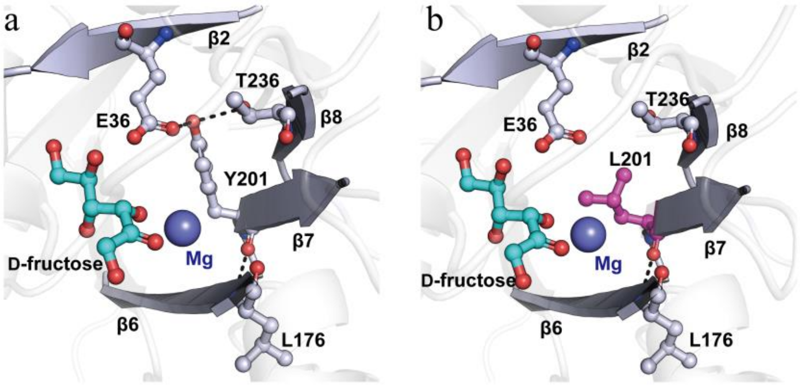

Figure S9. Distribution of the hydrogen bond network of the WT ADAE (a) and the variant M42 (b) in the substrate binding pocket. D-fructose was shown as cyan sticks, while Mg^{2+} was represented as a blue sphere. Hydrogen bonds were shown as black dashed lines.

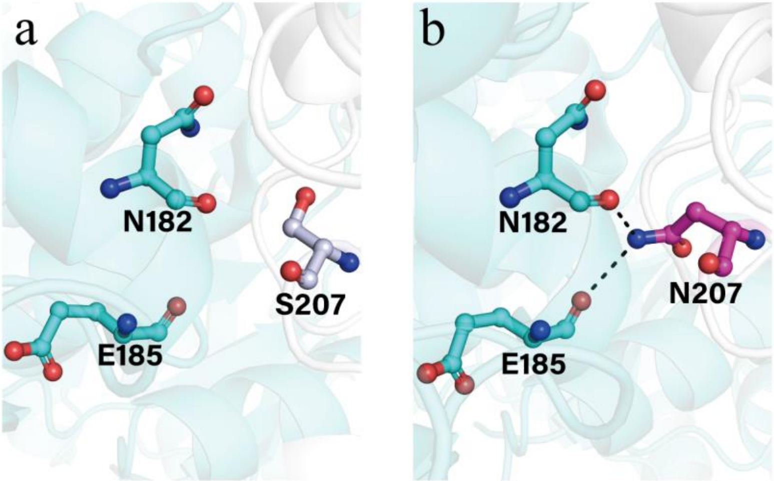

Figure S10. Inter-subunit interface of the region neighboring the residue at position 207 for the WT ADAE and the M42 variant. Chain A was shown in white and cyan for chain B. The residue Asn207 of chain A enables hydrogen bond formation with the residues Asn182 and Glu185 of chain B. The residues and hydrogen bonds were shown as sticks and black dashed lines, respectively.

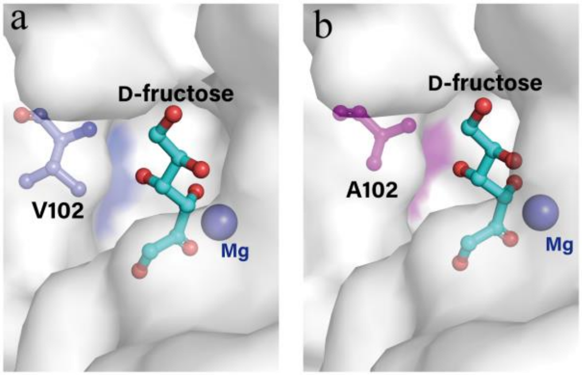

Figure S11. Structural comparison of the substrate binding pockets of WT ADAE (a) and the M42 variant (b). D-Fructose was shown as cyan sticks, while Mg^{2+} was represented as a blue sphere.

Table S1. Strains and plasmids used in this study.

Strains and plasmids	Description	Source
Strains		
E. coli JM109	Host for cloning plasmids	Lab stock
E. coli BL21	Host for expression plasmids	Lab stock
GBIO1	*E. coli* BL21 carrying plasmids pJ23109-psiR-pET22b and pPsiA-Cm^R-pET28a	This study
GBIO2	*E. coli* BL21 carrying plasmids pJ23105-psiR-pET22b and pPsiA-Cm^R-pET28a	This study
GBIO3	*E. coli* BL21 carrying plasmids pJ23106-psiR-pET22b and pPsiA-Cm^R-pET28a	This study
GBIO4	*E. coli* BL21 carrying plasmids pJ23104-psiR-pET22b and pPsiA-Cm^R-pET28a	This study
GBIO5	*E. coli* BL21 carrying plasmids pJ23100-psiR-pET22b and pPsiA-Cm^R-pET28a	This study
GBIO6	*E. coli* BL21 carrying plasmids pJ23104-psiR-pPsiA-gfp-pET22b and pPsiA-Cm^R-pET28a	This study
GBIO7	*E. coli* BL21 carrying plasmids pJ23104-psiR-pPsiA-gfp-pET22b and pJ23100-adae-pPsiA-Cm^R-pET28a	This study
GBIO8	*E. coli* BL21 carrying plasmids pJ23104-psiR-pPsiA-gfp-pET22b and pJ23105-adae-pPsiA-Cm^R-pET28a	This study

	E. coli BL21 carrying plasmids	
GBIO9	pJ23104-psiR-pPsiA-gfp-pET22b and pJ23106-adae-pPsiA-Cm^R-pET28a	This study
Plasmids		
pET-22b	Expression vector, Amp^R	Lab stock
pET-22b	Expression vector, Kan^R	Lab stock
pJ23109-psiR-pET22b	pET-22b harboring harboring the pJ23109-psiR expression cassette	This study
pJ23105-psiR-pET22b	pET-22b harboring the pJ23105-psiR expression cassette	This study
pJ23106-psiR-pET22b	pET-22b harboring the pJ23106-psiR expression cassette	This study
pJ23104-psiR-pET22b	pET-22b harboring the pJ23104-psiR expression cassette	This study
pJ23100-psiR-pET22b	pET-22b harboring the pJ23100-psiR expression cassette	This study
pJ23104-psiR-pPsiA-gfp-pET22b	pET-22b harboring the pJ23104-psiR and pPsiA-gfp expression cassettes	This study
pPsiA-Cm^R-pET28a	pET-28a harboring the pPsiA-Cm^R expression cassette	This study
pJ23100-adae-pPsiA-Cm^R-pET28a	pET-28a harboring the pJ23100-adae and pPsiA-Cm^R expression cassette	This study
pJ23105-adae-pPsiA-Cm^R-pET28a	pET-28a harboring the pJ23105-adae and pPsiA-Cm^R expression cassette	This study
pJ23106-adae-pPsiA-Cm^R-pET28a	pET-28a harboring the pJ23106-adae and pPsiA-Cm^R expression cassette	This study

Table S2. Primers used in this study.

Primers	Sequence of primer
For generation of site-saturation mutagenesis library of ADAE	
H7	F: 5'-GTGNNKGCGATGATGTGGAGCC-3'
	R: 5'-GCCAAAGCCTTGCATCATATGTATATCT-3'
M9	F: 5'-GCGNNKATGTGGAGCCTGAACTG-3'
	R: 5'-ATGCACGCCAAAGCCTTGCAT-3'
P38	F: 5'-ATTNNKCTGGTGGACCTGCCGAG-3'
	R: 5'-TTCAATAAAATCTTGGCCATAATCCGC-3'
L39	F: 5'-CCGNNKGTGGACCTGCCGA-3'
	R: 5'-AATTTCAATAAAATCTTGGCCATAATCCG-3'
V40	F: 5'-CTGNNKGACCTGCCGAGCGTG-3'
	R: 5'-CGGAATTTCAATAAAATCTTGGCCATAAT-3'
S64	F: 5'-TGCNNKTTAGTGTTACCGGAACCGG-3'
	R: 5'-CGCCGCGCGCAGGCCATATTTT-3'
L65	F: 5'-AGTNNKGTGTTACCGGAACCGGCGTGGGC-3'
	R: 5'-GCACGCCGCGCGCAGGCCATATTT-3'
V66	F: 5'-TTANNKTTACCGGAACCGGCGTG-3'
	R: 5'-TTANNKTTACCGGAACCGGCGTG-3'
V102	F: 5'-GGCNNKACCTATGGCGGCACGAGC-3'
	R: 5'-GGTCAGCGCTTCCGCGCCCATTT-3'
T107	F: 5'-GGCNNKAGCGAACGCACCGGCT-3'
	R: 5'-GCCATAGGTCACGCCGGTCAGCG-3'
V146	F: 5'-GCGNNKAACCGCTATGAAAACCATCTG-3'

	R: 5'-TTCAATGCCAAACTGCAGGCCCAG-3'
N147	F: 5'-GTGNNKCGCTATGAAAACCATCTGGTGAA-3'
	R: 5'-CGCTTCAATGCCAAACTGCAGGC-3'
H175	F: 5'-GTGNNKCTGGATACCTTTCACATGAACATG-3'
	R: 5'-AAAAATGTTATCCGCGCCAATGC-3'
F179	F: 5'-ACCNNKCACATGAACATGGAAGAAAAAGG-3'
	R: 5'-ATCCAGATGCACAAAAATGTTATCCGC-3'
Y201	F: 5'-AAANNKATGCACATGAGCGAAAGCG-3'
	R: 5'-CAGATAATCATGCGCCGCAATAATG-3'
S205	F: 5'-ATGNNKGAAAGCGACCGCGGCA-3'
	R: 5'-GTGCATATATTTCAGATAATCATGCGCCG-3'
T236	F: 5'-CTGNNKCTGGAGAGCTTTGCGGC-3'
	R: 5'-CACGCCTTTAAAGCCAATCGCC-3'
For generation of random mutagenesis library of ADAE	
ADAE-F	5'-GGAATTCCATATGATGCAAGGCTTTGGCGTGCAT-3'
ADAE-R	5'-CGGGATCCAAAAATGCGATACTGACTCGCTTTATCG-3'
For construction of the pPsiA-*Cm*^{R} gene fragment	
pPsiA-*Cm*^{R}-U1	5'-GGAATTCGTATAAATGGTGGCTTTTTTTGAACTTATGC-3'
pPsiA-*Cm*^{R}-U2	5'-ATTCCGTGGTTCAACGCCGTACAAATGGAGGAAAAGAGGAGAAAAATGAACTTTAATAAAAT-3'
pPsiA-*Cm*^{R}-D1	5'-ATTTTATTAAAGTTCATTTTTCTCCTCTTTTCCTCCATTTGTACGGCGTTGAACCACGGAAT-3'
pPsiA-*Cm*^{R}-D2	5'-CCGCTCGAGTTATAAAAGCCAGTCATTAGGCCTATCTGA-3'
For generation of the pPsiA-*gfp* gene fragment	
pPsiA-*gfp*-U1	5'-CCCAAGCTTGTATAAATGGTGGCTTTTTTTGAACTTATGCCC-3'

pPsiA-*gfp*-U2	5'-CCGTGGTTCAACGCCGTACAAATGGAGGAAAAGAGGAGAAAAATGGTTTCTAAAGGTGAAGA-3'
pPsiA-*gfp*-D1	5'-TCTTCACCTTTAGAAACCATTTTTCTCCTCTTTTCCTCCATTTGTACGGCGTTGAACCACGG-3'
pPsiA-*gfp*-D2	5'-CCGCTCGAGTTATTTGTACAGTTCGTCCATACCCAGGGTG-3'

Table S3. Data collection and refinement statistics.

	apo	FRU	PSJ
**Data Collection**			
Beamline	PF BL-1A	PF BL-1A	PF BL-1A
Wavelength (Å)	1.0800	1.0800	1.0800
Space group	$P2_12_12_1$	$P2_12_12_1$	$P2_12_12_1$
a, *b*, *c* (Å)	66.19, 97.95, 160.17	66.17, 97.52, 159.92	67.38, 98.71, 161.00
α, *β*, *γ* (°)	90.00, 90.00, 90.00	90.00, 90.00, 90.00	90.00, 90.00, 90.00
Resolution (Å)	46.88-2.05 (2.10-2.05)[a]	48.76-2.12 (2.18-2.12)[a]	47.19-2.32 (2.40-2.32)[a]
No. reflections	66,104 (4,343)	59,560 (4,561)	47,351 (4,570)
R_{meas}	0.097(1.161)	0.250(1.471)	0.184(1.594)
R_{pim}	0.037(0.430)	0.096(0.559)	0.051(0.437)
CC(1/2)[b]	0.999(0.750)	0.993(0.693)	0.998(0.712)
Mean I/σ(I)	14.1(1.9)	9.0(2.0)	11.1(2.0)
Completeness (%)	99.9(99.5)	100.0(100.0)	100.0(100.0)
Multiplicity	6.8(7.1)	6.7(6.8)	13.1(13.1)
**Refinement**			
Resolution (Å)	46.84-2.05	48.76-2.12	45.17-2.32
No. reflections	66015	59467	195270
R_{work}/[c]R_{free} (%)	17.7/22.5	18.3/22.9	16.9/18.8
No. atoms			
Protein	8580	8576	8720
Water	299	350	1091
Ligand	48	48	20
Metal	4	4	4
B-factors ($Å^2$)			
Protein	38.22	36.32	23.77
Water	37.90	36.14	32.79
Ligand	43.05	41.18	46.14
Metal	20.06	22.62	14.91
RMSD[d]			
Bond length (Å)	0.007	0.007	0.006
Bond angle (°)	0.835	0.859	0.857
Ramachandran plot (%)			
Favoured region	96.98	96.53	98.3
Allowed region	2.93	3.38	1.7

Outliers	0.09	0.09	0.0
PDB ID code	7ERM	7ERN	7ERO

[a] Values in parentheses are for the highest-resolution shell.

[b] CC 1/2; percentage of correlation between intensities from random half-datasets.

[c] R_{free} calculated with the 5% of reflections excluded from the refinement.

[d] RMSD; root-mean-square deviation.

Table S4. Specific activities and residual activity of ADAE and its variants toward D-fructose.

Emzymes	Mutation	Specific activity (U/mg)	Residual activity (%)
WT	None	44.5	32.6
M11	P38N	100.2	43.7
M12	V102A	73.4	34.5
M13	V102I	62.7	41.8
M14	T107N	81.8	19.6
M15	Y201L	122.8	40.6
M16	Y201V	72.9	42.3
M17	T236K	58.7	19.7
M21	P38N/Y201L	172.2	49.6
M22	P38N/T107N/Y201L	103.2	40.9
M23	P38N/V102A/Y201L	201.1	46.6
M24	P38N/V102A/Y201L/T236K	154.4	38.1
M31	P38N/T100E/V102A/Y201L	215.8	45.3
M32	P38N/V102A/Y201L/S207N	197.1	79.9

M33	P38N/V102A/Y201L/I251R	286.6	49.4
M41	P38N/T100E/V102A/Y201L/I251R	231.8	44.9
M42	P38N/V102A/Y201L/S207N/I251R	279.5	75.7

References

[1] H. M. Qin, D. Gao, M. Zhu, C. Li, Z. Zhu, H. Wang, W. Liu, M. Tanokura, F. Lu, *Int. J. Biol. Macromol.* **2020**, *147*, 1309-1317.

[2] C. Li, X. Gao, H. Qi, W. Zhang, L. Li, C. Wei, M. Wei, X. Sun, S. Wang, L. Wang, Y. Ji, S. Mao, Z. Zhu, M. Tanokura, F. Lu, H. M. Qin, *Angew. Chem. Int. Ed.* **2023**, *62*, e202216721.

[3] P. Emsley, B. Lohkamp, W. G. Scott, K. Cowtan, *Acta. Crystallogr. D. Biol. Crystallogr.* **2010**, *66*, 486-501.

[4] P. D. Adams, P. V. Afonine, G. Bunkóczi, V. B. Chen, I. W. Davis, N. Echols, J. J. Headd, L. W. Hung, G. J. Kapral, R. W. Grosse-Kunstleve, A. J. McCoy, N. W. Moriarty, R. Oeffner, R. J. Read, D. C. Richardson, J. S. Richardson, T. C. Terwilliger, P. H. Zwart, *Acta. Crystallogr. D. Biol. Crystallogr.* **2010**, *66*, 213-221.
